# Clinicopathological and Perioperative Factors Associated with Overall Survival and 90-Day Mortality Following Esophagectomy for Esophageal Cancer

**DOI:** 10.3390/jcm15197453

**Published:** 2026-09-24

**Authors:** Hüseyin Esin, Murat Güner

**Affiliations:** 1 Department of General Surgery, Izmir City Hospital, University of Health Sciences, 35540 Izmir, Türkiye; 2 Department of Surgical Oncology, Izmir City Hospital, University of Health Sciences, 35540 Izmir, Türkiye

**Keywords:** esophageal cancer, esophagectomy, overall survival, 90-day mortality, lymph node metastasis, neoadjuvant chemotherapy, tumor regression grade

## Abstract

**Background/Objectives:** Esophageal cancer (EC) is an aggressive malignancy, and outcomes following esophagectomy are influenced by both oncological and perioperative factors. This study aimed to identify clinicopathological and perioperative factors associated with overall survival (OS) after esophagectomy for EC and, secondarily, to evaluate factors associated with 90-day mortality. **Methods:** A total of 101 patients who underwent radical esophagectomy with two-field lymphadenectomy between April 2016 and April 2023 were retrospectively evaluated. The primary outcome was OS, defined as the time from esophagectomy to death from any cause or the date of last follow-up. The secondary outcome was 90-day postoperative mortality. OS was evaluated using Kaplan–Meier and Cox proportional hazards analyses, whereas 90-day mortality was assessed using Firth penalized-likelihood logistic regression because of the limited number of events. **Results:** During follow-up, 74 patients (73.3%) died, including 11 deaths within 90 days after esophagectomy. The estimated 1-, 3-, and 5-year OS rates were 80.1%, 44.8%, and 38.0%, respectively. In the whole-cohort multivariable Cox model, stratified by surgical approach, lymph node positivity was associated with poorer OS (HR 10.57, 95% CI 4.78–23.38; *p* < 0.001). Among patients receiving neoadjuvant chemotherapy (n = 76), lymph node positivity (HR 6.34, 95% CI 2.60–15.45; *p* < 0.001) and pathological non-response (HR 2.96, 95% CI 1.13–7.73; *p* = 0.027) were associated with poorer OS. In exploratory Firth regression analysis, lymph node positivity (OR 103.11, 95% CI 7.40 –> 1000; *p* < 0.001) and intraoperative complications (OR 75.58, 95% CI 7.42 –> 1000; *p* < 0.001) were strongly associated with 90-day mortality. **Conclusions:** Lymph node positivity was consistently associated with poorer OS both in the entire cohort and among patients receiving neoadjuvant chemotherapy. Pathological non-response was additionally associated with poorer OS in the neoadjuvant chemotherapy subgroup, while lymph node positivity and intraoperative complications were strongly associated with 90-day mortality.

## 1. Introduction

Esophageal cancer (EC) is an aggressive gastrointestinal malignancy and remains a major global health concern. According to GLOBOCAN 2024 estimates, approximately 494,000 new cases and 442,000 deaths occur worldwide annually, making EC the 13th most commonly diagnosed cancer and the seventh leading cause of cancer-related mortality globally [1].

The management of EC requires a multidisciplinary approach, with surgery, systemic therapy, and radiotherapy constituting the principal treatment modalities. Endoscopic resection is recommended for selected patients with superficial or early-stage esophageal neoplasia, whereas multimodality treatment incorporating preoperative chemoradiotherapy or perioperative systemic chemotherapy in combination with surgery is recommended for appropriately selected patients with resectable locally advanced esophageal or esophagogastric junction cancer [2,3,4].

Esophagectomy remains an important component of curative-intent treatment in appropriately selected patients. Lymph node metastasis, particularly in esophageal adenocarcinoma, represents one of the most powerful prognostic indicators of survival [5,6]. Patterns of lymphatic spread are heterogeneous and are influenced by several factors, including tumor location, histological subtype, T category, and the administration of neoadjuvant therapy (NT) [7].

Serum tumor markers, including carcinoembryonic antigen (CEA) and carbohydrate antigen 19-9 (CA19-9), have been investigated as potential adjunctive prognostic biomarkers in EC; however, their clinical utility remains limited [8,9]. Evidence regarding the prognostic value of serum cancer antigen 15-3 (CA15-3) in EC remains scarce, and the limited available data have not demonstrated a significant association between preoperative CA15-3 levels and overall survival following esophagectomy [10]. In the present retrospective study, CA15-3 was one of several preoperative laboratory variables available in the medical records, and its potential association with overall survival and 90-day postoperative mortality was therefore evaluated on an exploratory basis alongside other clinical, pathological, and perioperative variables.

The primary aim of this study was to evaluate overall survival (OS) following esophagectomy for EC and to identify clinical, pathological, and perioperative factors associated with overall survival. As a secondary objective, we evaluated factors associated with 90-day mortality.

## 2. Materials and Methods

### 2.1. Study Design, Ethical Approval, and Patient Selection

This retrospective cohort study was conducted in accordance with the principles of the Declaration of Helsinki and was approved by the Ethics Committee of the University of Health Sciences, Tepecik Training and Research Hospital (approval no. 2023/04-11; 3 May 2023). This study included patients who underwent radical esophagectomy for EC at our institution between April 2016 and April 2023. During the study period, 136 patients were screened for eligibility. Thirty-five patients were excluded: 17 because the tumor was found to be unresectable at surgical exploration, 10 because of insufficient clinical or pathological data, and 8 because of loss to follow-up. The final study cohort therefore comprised 101 patients who underwent radical esophagectomy with two-field lymphadenectomy.

Patients with very early EC (cTis–cT1a) treated by endoscopic resection were not part of the surgical cohort and were therefore not included among the screened patients. Similarly, patients with cervical EC treated with definitive chemoradiotherapy were not considered candidates for esophagectomy and were not included in the screened cohort. The patient-selection process is shown in Figure 1.

### 2.2. Preoperative Evaluation, Staging, and Treatment Strategy

Diagnosis was confirmed by upper gastrointestinal endoscopy in all patients. Preoperative evaluation included complete blood count, biochemical tests, and thoracoabdominal computed tomography. Magnetic resonance imaging and/or fluorodeoxyglucose positron emission tomography/computed tomography was additionally performed when clinically indicated, particularly in patients with suspected liver lesions or distant metastatic disease.

Clinical staging and treatment planning were based on available preoperative imaging and multidisciplinary tumour board assessment. Tumour, node, and metastasis staging was performed according to the 8th edition of the American Joint Committee on Cancer (AJCC) TNM classification [11]. Pretreatment clinical staging was designated as cTNM, whereas postoperative pathological staging was designated as pTNM in patients undergoing upfront esophagectomy and as ypTNM in patients undergoing esophagectomy following NT [11].

Patients with cT1bN0 disease were considered for upfront esophagectomy, whereas patients with resectable locally advanced disease were evaluated for NT followed by surgery. All patients categorized as T1a in the postoperative pathological staging analyses had received NT and were classified as ypT1a on final histopathological examination; no patient with cT1a disease underwent esophagectomy.

Three patients with high-risk cT2 disease for whom NT was recommended declined NT and underwent upfront esophagectomy.

### 2.3. Neoadjuvant Treatment and Assessment of Response

Neoadjuvant chemotherapy was administered to 76 patients (75.2%), of whom 53 received concurrent or sequential radiotherapy as part of a chemoradiotherapy regimen. The remaining 25 patients (24.8%) underwent upfront surgery. NT strategies were consistent with the contemporaneous National Comprehensive Cancer Network (NCCN) recommendations for esophageal and esophagogastric junction cancers applicable during the study period [2,3,12] and were determined within the institutional multidisciplinary treatment framework. These recommendations support multimodal treatment approaches in appropriately selected patients with resectable locally advanced disease and emphasize multidisciplinary treatment planning [2,3,12].

Pathological response to NT was assessed on the resection specimen using the five-tier Mandard tumour regression grade (TRG) system: TRG 1, complete regression with no residual viable tumour cells; TRG 2, rare residual tumour cells scattered within fibrosis; TRG 3, residual tumour with fibrosis predominating over tumour cells; TRG 4, residual tumour predominating over fibrosis; and TRG 5, no evidence of tumour regression [13]. For the purposes of analysis, patients with TRG 1–2 were classified as responders and those with TRG 3–5 as non-responders. Pathological complete response (pCR) was defined as the absence of residual viable tumour in both the primary tumour and regional lymph nodes (ypT0N0) [14].

### 2.4. Surgical Technique and Perioperative Management

For patients who received neoadjuvant treatment, esophagectomy was generally scheduled approximately 4–6 weeks after completion of treatment, in accordance with institutional practice during the study period [4,15]. The choice between Ivor Lewis and McKeown esophagectomy was based primarily on tumour location, patient characteristics, and surgeon preference. Anastomotic techniques were categorized as hand-sewn or stapled for statistical analysis. All hand-sewn anastomoses were cervical anastomoses performed during McKeown esophagectomy (n = 21). Among the stapled anastomoses, 75 were intrathoracic anastomoses performed during Ivor Lewis esophagectomy and 5 were cervical anastomoses performed during McKeown esophagectomy. All intrathoracic anastomoses were constructed using a side-to-side linear stapled technique. Surgical approaches were classified into three categories according to the operative technique ultimately used: totally minimally invasive esophagectomy, in which the abdominal and thoracic phases were performed laparoscopically and thoracoscopically, respectively (n = 72, 71.3%); hybrid esophagectomy, consisting of a laparoscopic abdominal phase combined with open thoracotomy (n = 12, 11.9%); and open esophagectomy, in which both phases were performed using an open approach (n = 17, 16.8%). Conversion to an open approach was required in six patients because of inadequate visualization or intraoperative complications. Converted procedures were classified according to the operative approach ultimately completed.

Gastric conduit reconstruction was performed in all patients. Feeding jejunostomy was placed selectively in patients considered to be at increased nutritional risk, and pyloromyotomy was not routinely performed. Thoracic and abdominal drains were placed at the completion of surgery. The abdominal drain was generally removed on postoperative day 2 or 3. Thoracic drain removal was individualized according to clinical criteria, including the absence of an air leak or chylous drainage, acceptable daily drainage volume, and satisfactory lung expansion on chest imaging.

### 2.5. Pathological Evaluation and Follow-Up

Pathological findings were reviewed by the multidisciplinary oncology board to guide postoperative management. Follow-up visits were scheduled at 1, 3, and 6 months after surgery and at 6-month intervals thereafter. During follow-up, locoregional recurrence and distant metastasis were assessed based on available clinical and radiological follow-up data.

### 2.6. Study Variables and Outcome Definitions

The following variables were retrospectively evaluated: age, sex, smoking and comorbidities, American Society of Anesthesiologists (ASA) score, preoperative laboratory parameters, serum tumour markers (CEA, CA15-3, and CA19-9), tumour histological subtype and location, postoperative pathological T category, lymph-node status, resection-margin status, neoadjuvant and postoperative oncological treatment, operative procedure, surgical approach (totally minimally invasive, hybrid, or open), operative time, anastomotic technique (stapled or hand-sewn), intraoperative complications, and postoperative complications, including wound infection, anastomotic leakage, cardiopulmonary events, and other systemic complications. Anastomotic stricture and locoregional recurrence were also recorded. Serum CEA, CA15-3, and CA19-9 levels were measured as part of the routine preoperative clinical assessment. Locoregional recurrence was defined as recurrence at the primary tumour/anastomotic site or within regional lymph-node basins during postoperative follow-up.

The primary outcome of the study was OS, defined as the time from esophagectomy to death from any cause or the date of last follow-up. The secondary outcome was 90-day mortality, defined as death from any cause within 90 days after esophagectomy. Overall mortality was additionally reported descriptively as the proportion of patients who died from any cause during the entire follow-up period.

### 2.7. Statistical Analysis

The distribution of continuous variables was assessed using the Kolmogorov–Smirnov and Shapiro–Wilk tests, together with graphical assessment where appropriate. Normally distributed continuous variables were summarized as mean ± standard deviation, whereas non-normally distributed variables were presented as median with the corresponding range or interquartile range, as appropriate. Categorical variables were summarized as frequencies (n) and percentages (%). Comparisons of continuous variables between two independent groups were performed using the independent-samples t test for normally distributed variables and the Mann–Whitney U test for non-normally distributed variables. Categorical variables were compared using Pearson’s chi-square test or Fisher’s exact test, as appropriate.

Postoperative 90-day mortality was analyzed as a binary outcome. Because sparse-event data and separation may lead to biased or unstable estimates with conventional maximum-likelihood logistic regression, associations with 90-day mortality were evaluated using Firth penalized-likelihood logistic regression. Results from these models were expressed as odds ratios (ORs) with profile-penalized-likelihood 95% confidence intervals and penalized likelihood-ratio *p* values. Given the limited number of outcome events (11 deaths), the adjusted model was restricted to two clinically relevant covariates to reduce the risk of overfitting, and the resulting estimates were considered exploratory.

OS was analyzed using the Kaplan–Meier method, and 1-, 3- and 5-year survival estimates were reported with 95% confidence intervals (CI) together with the number of patients at risk. Differences between survival distributions were assessed using the log-rank (Mantel–Cox) test.

Associations between candidate prognostic variables and OS were evaluated using Cox proportional hazards regression and reported as hazard ratios (HRs) with 95% confidence intervals. Candidate variables for multivariable modeling were selected based on clinical relevance and their univariable associations with OS, while also considering the temporal relationship of each variable to the survival outcome and the number of observed events. Variables representing perioperative or follow-up events, including intraoperative complications, anastomotic leakage, locoregional recurrence, and distant metastasis, were not included as baseline prognostic covariates in the final multivariable Cox models. Intraoperative complications were evaluated separately in the analysis of 90-day mortality.

Two multivariable Cox models were constructed. The primary whole-cohort model included lymph node status and ASA score and was stratified by surgical approach because surgical approach did not satisfy the proportional hazards assumption. A separate model was constructed for patients who received neoadjuvant chemotherapy (n = 76), irrespective of the addition of radiotherapy, and included NT response, ypT category, lymph node status, and ASA score. The proportional hazards assumption was assessed using scaled Schoenfeld residuals and was satisfied for the covariates retained in the final models. Model discrimination was assessed using Harrell’s concordance index (c-index). Median follow-up duration was estimated using the reverse Kaplan–Meier method. All statistical analyses were performed using SPSS software version 30.0 (IBM Corp., Armonk, NY, USA), and R software version 4.5.1 (R Foundation for Statistical Computing, Vienna, Austria), with two-sided *p* < 0.05 considered statistically significant.

## 3. Results

The cohort consisted predominantly of males (57.4%), and 61.4% of patients were aged 65 years or older. During the entire follow-up period, 74 patients (73.3%) died from any cause, including 11 patients who died within 90 days after esophagectomy. Patients with 90-day mortality were older and were more frequently male, and had higher rates of smoking, ASA III–IV status, hypertension, and coronary artery disease (all *p* < 0.05, Table 1). In descriptive comparisons of overall mortality, patients who died during follow-up had higher rates of smoking and ASA III–IV status and more frequently had elevated CA15-3 levels than those who were alive at the last follow-up. Demographic and baseline characteristics are summarized in Table 1.

Patients with 90-day mortality had higher rates of intraoperative complications, advanced pathological T category, lymph node positivity, and positive surgical margins than those without 90-day mortality (all *p* < 0.01). In descriptive comparisons of overall mortality, the distribution of surgical approach and anastomotic technique differed according to mortality status, while lymph node positivity and advanced pathological T category were more frequent among patients who died during follow-up (all *p* < 0.05). Among patients who developed distant metastasis during follow-up, the liver was the most common metastatic site (58.1%), followed by the lung (27.9%) and peritoneal or multiple sites (14.0%). Surgical and oncological characteristics are summarized in Table 2.

Seventy-two patients (71.3%) underwent totally minimally invasive esophagectomy, 12 (11.9%) underwent a hybrid procedure combining a laparoscopic abdominal phase with thoracotomy, and 17 (16.8%) underwent open esophagectomy. All 11 patients with 90-day mortality were in the hybrid group, whereas no 90-day deaths occurred in the totally minimally invasive or open groups (*p* < 0.001). In descriptive comparisons of overall mortality, mortality rates were 76.4% (55/72), 91.7% (11/12), and 47.1% (8/17) in the totally minimally invasive, hybrid, and open groups, respectively (*p* = 0.027, Table 2).

Anastomotic leakage, analyzed separately from anastomotic stricture, occurred in six patients (5.9%). Five of the 11 patients with 90-day mortality (45.5%) had an anastomotic leak, compared with one of the 90 patients without 90-day mortality (1.1%; *p* < 0.001, Table 2). All six patients with anastomotic leakage died during follow-up, compared with 68 of 95 patients without leakage (100% vs. 71.6%); however, this difference was not statistically significant in the descriptive overall mortality comparison (*p* = 0.189). Leakage occurred in 5 of 50 smokers (10.0%) and 1 of 51 non-smokers (2.0%), although this difference was not statistically significant (*p* = 0.112). In time-to-event analysis, anastomotic leakage was also associated with markedly shorter OS, as detailed below.

Among the 76 patients who received neoadjuvant chemotherapy, ypT category differed significantly according to 90-day mortality status (*p* < 0.001, Table 2). No 90-day deaths occurred among patients with ypT0–ypT1b disease, whereas five of the seven patients with ypT4a disease died within 90 days. Overall mortality also increased across advancing ypT categories in descriptive comparisons (*p* < 0.001, Table 2).

Neoadjuvant chemotherapy was administered to 76 patients (75.2%), whereas 25 patients (24.8%) underwent primary resection. The rate of 90-day mortality did not differ significantly according to the administration of neoadjuvant chemotherapy (*p* = 0.061, Table 2). Similarly, the rate of neoadjuvant chemotherapy did not differ significantly between patients who died during follow-up and those who were alive at the last follow-up (*p* = 0.493). Among the 76 patients who received neoadjuvant chemotherapy, 31 (40.8%) were classified as responders (TRG 1–2) and 45 (59.2%) as non-responders (TRG 3–5); the 25 patients who underwent primary resection were excluded from this analysis. All 11 patients with 90-day mortality were non-responders (*p* = 0.002, Table 2). Overall mortality was higher among non-responders than responders (40/45 [88.9%] vs. 17/31 [54.8%], *p* < 0.001, Table 2).

In Firth penalized-likelihood logistic regression, lymph node positivity and intraoperative complications showed strong associations with 90-day mortality in univariable analyses (Table 3). Given the limited number of events (n = 11), the bivariable model was restricted to these two clinically relevant variables. Both lymph node positivity (OR: 103.11, 95% CI 7.40–>1000; *p* < 0.001) and intraoperative complications (OR: 75.58, 95% CI 7.42–>1000; *p* < 0.001) remained strongly associated with 90-day mortality (Table 3). These findings should be interpreted as exploratory because of the small number of events.

In the multivariable Cox proportional hazards analysis of the entire cohort (Model 1), stratified by surgical approach because of violation of the proportional hazards assumption, lymph node positivity was strongly associated with poorer OS (HR: 10.57, 95% CI 4.78–23.38; *p* < 0.001), whereas ASA III–IV status was not significantly associated with OS (HR: 1.38, 95% CI 0.83–2.28; *p* = 0.214) (Table 4).

In the subgroup analysis of patients who received neoadjuvant chemotherapy (n = 76; Model 2), non-response to treatment was associated with poorer OS after adjustment for ypT category, lymph node status, and ASA score (HR: 2.96, 95% CI 1.13–7.73; *p* = 0.027, Table 4). Lymph node positivity was also associated with poorer OS (HR: 6.34, 95% CI 2.60–15.45; *p* < 0.001), whereas ypT category and ASA III–IV status were not statistically significant (Table 4). The concordance index was 0.73 for Model 1 and 0.82 for Model 2 (Table 4).

Kaplan–Meier analysis of the entire cohort demonstrated estimated 1-, 3-, and 5-year OS rates of 80.1% (95% CI, 72.6–88.3%), 44.8% (95% CI, 35.6–56.5%), and 38.0% (95% CI, 28.9–50.0%), respectively, with a median OS of 28 months (95% CI, 23–54) (Figure 2).

OS differed significantly according to lymph node status (log-rank *p* < 0.001). Patients with lymph node-positive disease had a median OS of 13 months (95% CI, 7–17), whereas those with lymph node-negative disease had a median OS of 66 months (95% CI, 41–73) (Figure 3). Patients who developed anastomotic leakage also had markedly shorter OS than those without leakage (median OS, 1 vs. 31 months; log-rank *p* < 0.001) (Figure 4).

Among patients who received neoadjuvant chemotherapy, responders (TRG 1–2) had significantly better OS than non-responders (TRG 3–5), with median OS of 64 and 16 months, respectively (log-rank *p* < 0.001) (Figure 5). Survival also differed significantly across individual tumour regression grades (log-rank *p* < 0.001), demonstrating progressively poorer survival with increasing TRG (Figure 6).

Additional Kaplan–Meier analyses are presented in the Supplementary Materials. OS did not differ significantly according to smoking status (log-rank *p* = 0.073; Figure S1), whereas significant differences were observed according to surgical approach (log-rank *p* < 0.001; Figure S2), ypT category among patients receiving neoadjuvant chemotherapy (log-rank *p* < 0.001; Figure S3), and receipt of neoadjuvant chemotherapy (log-rank *p* = 0.004; Figure S4). Within the neoadjuvant chemotherapy subgroup, OS was also significantly poorer in patients with lymph node-positive disease than in those with lymph node-negative disease (log-rank *p* < 0.001; Figure S5). Detailed Kaplan–Meier estimates are provided in Table 5.

## 4. Discussion

In this retrospective cohort of patients undergoing esophagectomy for EC, lymph node positivity was the most consistent factor associated with poorer OS, both in the entire cohort and among patients who received neoadjuvant chemotherapy. In the neoadjuvant chemotherapy subgroup, pathological non-response was additionally associated with poorer OS after adjustment for ypT category, lymph node status, and ASA score. In the exploratory analysis of 90-day mortality, lymph node positivity and intraoperative complications showed strong associations with early postoperative death. These findings highlight the prognostic importance of oncological disease burden and pathological treatment response, as well as the contribution of perioperative events to early postoperative outcomes.

The relationship between perioperative outcomes and subsequent survival after esophagectomy has been demonstrated in large population-based studies. In a Dutch nationwide cohort of more than 7000 patients, van der Werf et al. reported that achievement of a “textbook outcome,” incorporating complete resection, adequate lymph node retrieval, and an uncomplicated postoperative course, was associated with improved conditional survival [16]. Although our observational design does not permit causal inference, these findings support the importance of both perioperative risk reduction and oncological quality of surgery.

Age and physiological reserve are important considerations in patients undergoing major oncological surgery. Previous studies have reported increased postoperative morbidity and mortality in older patients undergoing esophagectomy and emphasized careful patient selection and preoperative risk assessment [17,18,19,20]. In our cohort, however, age was not retained as a principal factor in the final multivariable OS model, and ASA score was not significantly associated with OS after adjustment for lymph node status.

The demographic and histopathological characteristics of our cohort were broadly consistent with established epidemiological patterns of EC, including male predominance and the relatively high frequency of squamous cell carcinoma [1,21,22]. These characteristics primarily describe the study population and were not among the principal factors associated with OS in the final models.

Smoking has been associated with adverse postoperative outcomes, including anastomotic leakage after esophagectomy [23,24,25,26]. In our cohort, anastomotic leakage was numerically more frequent among smokers, although this difference was not statistically significant. Similarly, smoking was not significantly associated with OS. Thus, our findings do not establish a significant association between smoking and either anastomotic leakage or survival.

Anastomotic leakage was strongly associated with 90-day postoperative mortality and was also associated with markedly poorer OS in Kaplan–Meier analysis. Although these findings indicate adverse outcomes among patients who developed leakage, the small number of leakage events limits the precision of the estimates and warrants cautious interpretation.

A surgical approach may influence perioperative outcomes after esophagectomy, and previous studies have reported potential advantages of minimally invasive and hybrid approaches [27,28], while emphasizing the importance of surgical experience and the learning curve [29,30]. In our cohort, surgical approach was associated with OS in unadjusted analysis but did not satisfy the proportional hazards assumption and was therefore used as a stratification factor in the whole-cohort Cox model rather than interpreted as an independent prognostic factor. Intraoperative complications, in contrast, were strongly associated with 90-day mortality in Firth regression, although the limited number of events warrants cautious interpretation.

The optimal anastomotic technique remains controversial, and studies comparing hand-sewn and stapled techniques have yielded heterogeneous findings [31,32,33,34]. In our cohort, anastomotic technique was not a principal variable in the final multivariable survival models. Given its non-random selection and relationship with operative approach and anastomotic location, our findings should not be interpreted as evidence of superiority of either technique.

Pathological disease burden remains a major determinant of prognosis following esophagectomy, with lymph node involvement being a well-established prognostic factor in EC [35]. In our study, lymph node positivity was consistently associated with poorer OS both in the entire cohort and among patients receiving neoadjuvant chemotherapy. Kaplan–Meier analysis showed the same pattern, with substantially poorer survival in patients with lymph node-positive disease. The consistency of this association across analyses reinforces the prognostic importance of residual nodal disease following esophagectomy.

NT is an integral component of multimodal management for resectable locally advanced EC [36], and pathological response has been associated with prognosis [37]. In our cohort, responders (TRG 1–2) had substantially better OS than non-responders (TRG 3–5), and pathological non-response remained associated with poorer OS after multivariable adjustment in patients receiving neoadjuvant chemotherapy. Survival also differed across individual TRG categories, further supporting pathological treatment response as an important prognostic feature.

Previous studies have compared multimodal strategies, including neoadjuvant chemoradiotherapy followed by surgery and definitive chemoradiotherapy [38,39]. Our study was not designed to compare these strategies, and the unadjusted difference in OS according to receipt of neoadjuvant chemotherapy should not be interpreted as a treatment effect because of differences in disease characteristics and treatment selection. Our findings are therefore more appropriately interpreted in terms of pathological response among treated patients.

Recurrence and distant metastasis remain important manifestations of disease progression after esophagectomy, with substantial recurrence rates reported despite multimodal treatment [40,41,42,43]. In our cohort, both recurrence and distant metastasis were frequently observed during follow-up. Because these events occurred after surgery, they were not incorporated as fixed baseline prognostic covariates in the final Cox models. Margin positivity was uncommon; nevertheless, achieving an R0 resection remains an important oncological objective [44].

Serum CA15-3 was evaluated exploratorily in this study. CA15-3 detects circulating epitopes associated with MUC1, which has been implicated in tumour progression and prognosis in several malignancies, including esophageal squamous cell carcinoma [45,46,47,48,49]. Associations between tissue MUC1 expression and prognosis have also been reported in gastrointestinal and breast malignancies [50,51]. In our cohort, elevated preoperative CA15-3 was more frequent among patients who died during follow-up in the descriptive analysis. However, this descriptive association is insufficient to establish a prognostic role for CA15-3. Moreover, tissue MUC1 expression was not assessed, and no direct relationship between serum CA15-3 and tumour MUC1 expression can be inferred. Therefore, our CA15-3 findings should be considered exploratory and require prospective validation.

Several limitations should be acknowledged. The retrospective, single-center design introduces potential selection and information bias and limits generalizability. The relatively small sample size, particularly the limited number of 90-day deaths, restricted the complexity and precision of multivariable modeling; therefore, the Firth regression findings should be considered exploratory. Surgical approach and anastomotic technique were not randomized and may have been influenced by patient and tumour characteristics, surgeon preference, and changes in practice over time. Detailed information regarding specific NT regimens was not consistently available. Finally, recurrence and distant metastasis were not incorporated as fixed baseline prognostic covariates because they occurred during follow-up. Larger prospective multicenter studies are warranted to validate these findings.

## 5. Conclusions

In this retrospective cohort, lymph node positivity was consistently associated with poorer OS after esophagectomy, both in the entire cohort and among patients receiving neoadjuvant chemotherapy. Pathological non-response was additionally associated with poorer OS in the neoadjuvant chemotherapy subgroup, while lymph node positivity and intraoperative complications were strongly associated with 90-day mortality in exploratory analysis. These findings highlight the prognostic relevance of nodal disease and pathological treatment response after esophagectomy. Given the retrospective design and the limited number of 90-day deaths, these findings, particularly those from the 90-day mortality analysis, should be interpreted cautiously and validated in larger prospective cohorts.

## Figures and Tables

**Figure 1:**
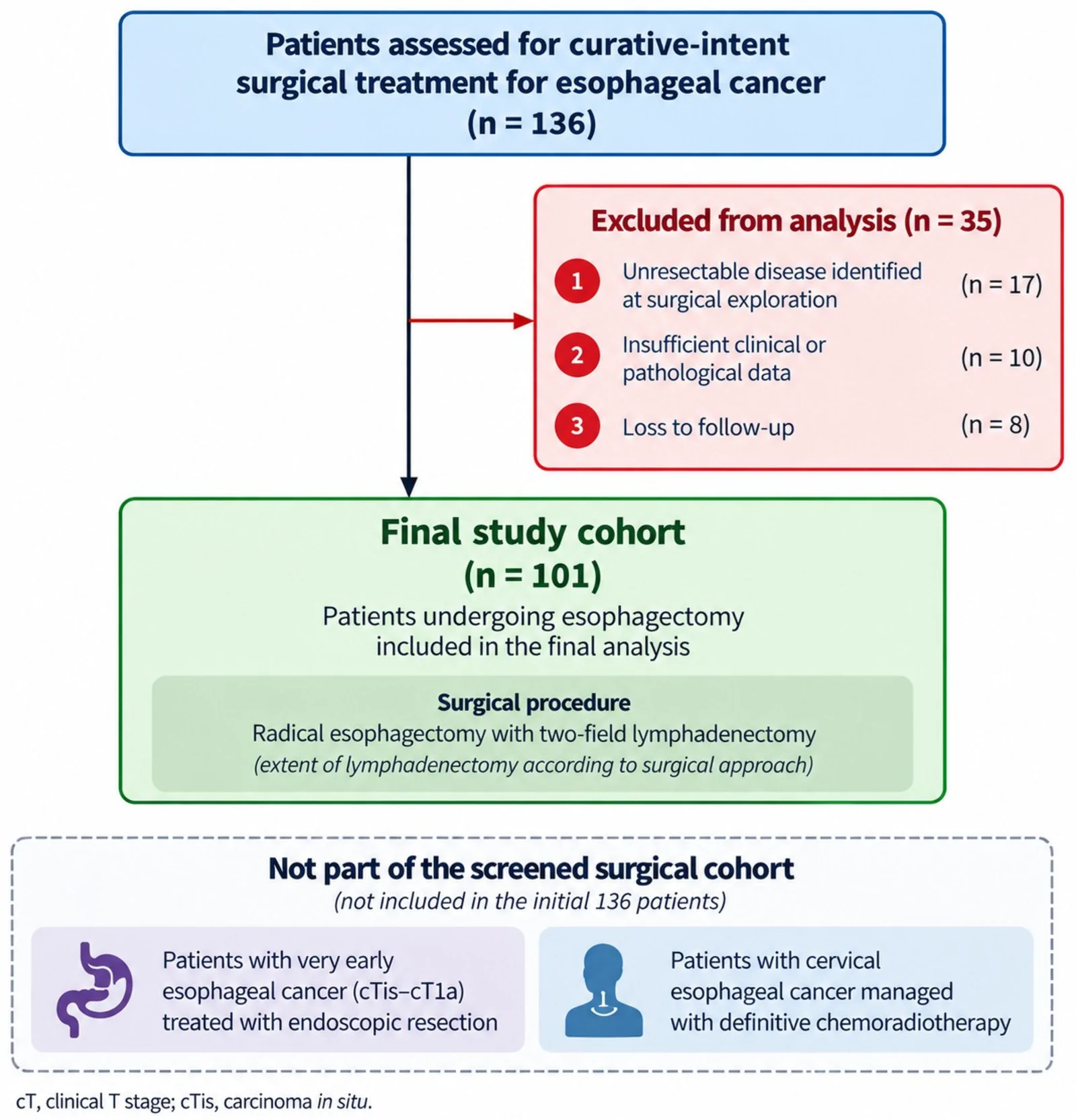
Flow diagram of patient selection and study cohort formation. A total of 136 patients assessed for curative-intent surgical treatment for esophageal cancer were screened, of whom 35 were excluded (17 because of unresectable disease identified at surgical exploration, 10 because of insufficient clinical or pathological data, and 8 because of loss to follow-up). The final study cohort comprised 101 patients who underwent radical esophagectomy with two-field lymphadenectomy. Patients with very early esophageal cancer (cTis–cT1a) treated with endoscopic resection and patients with cervical esophageal cancer managed with definitive chemoradiotherapy were not part of the screened surgical cohort.

**Figure 2:**
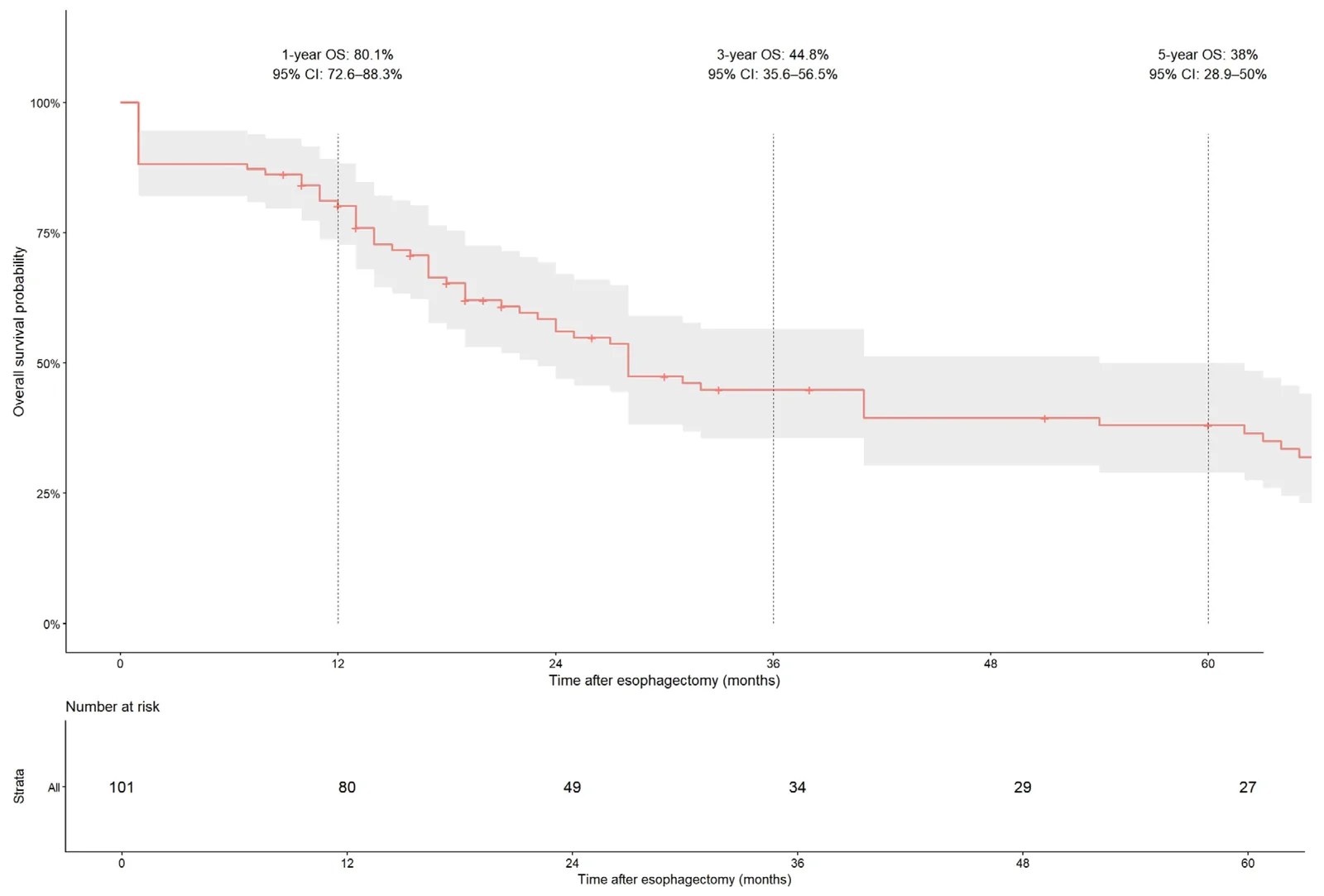
Kaplan–Meier curve for overall survival in the entire cohort. Estimated 1-, 3-, and 5-year OS rates were 80.1%, 44.8%, and 38.0%, respectively. Shaded areas represent 95% confidence intervals, and numbers at risk are shown below the survival curve.

**Figure 3:**
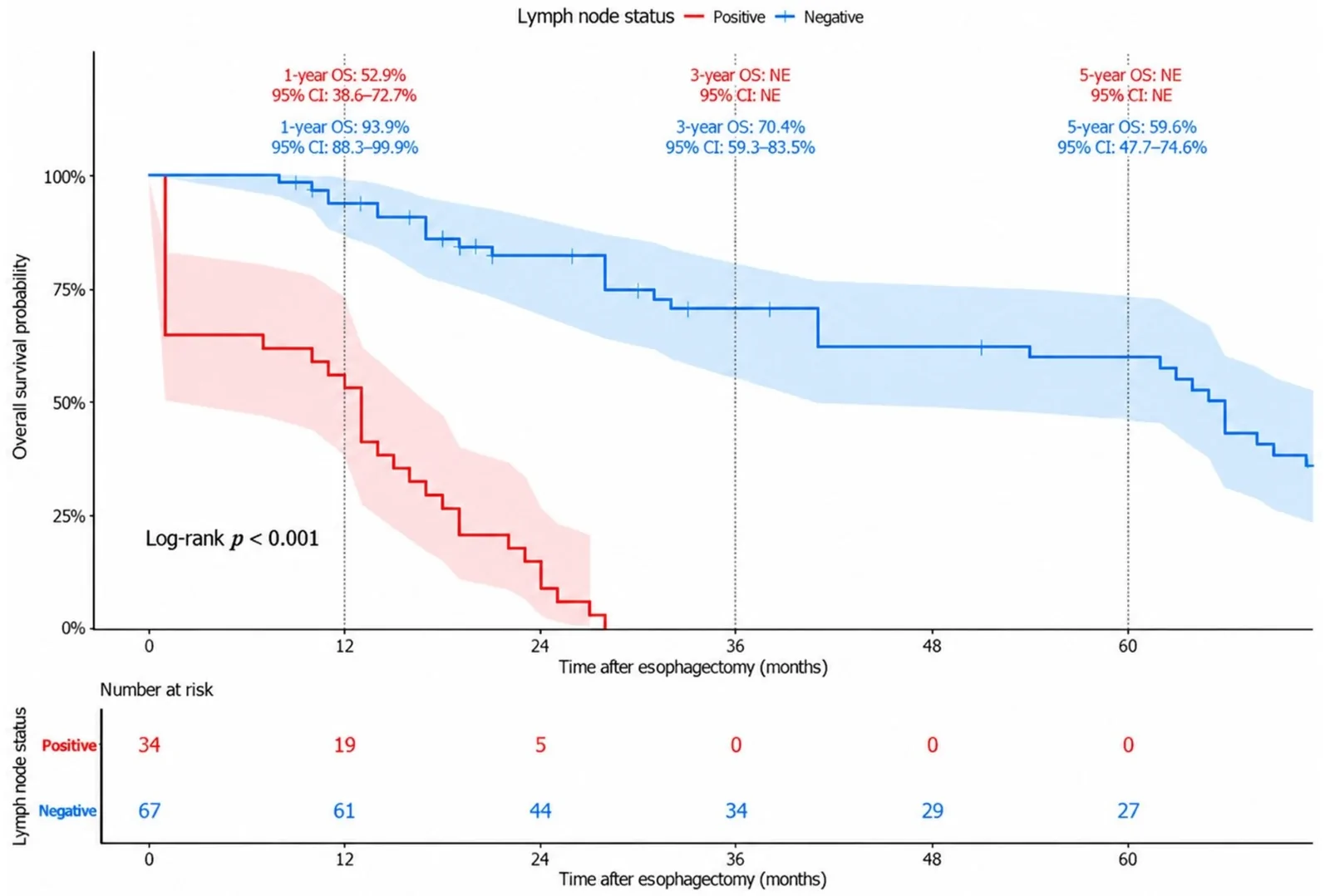
Kaplan–Meier overall survival curves according to lymph node status in the entire cohort. Overall survival was significantly poorer in patients with lymph node-positive disease than in those with lymph node-negative disease (log-rank *p* < 0.001). Shaded areas represent 95% confidence intervals, and numbers at risk are shown below the survival curves.

**Figure 4:**
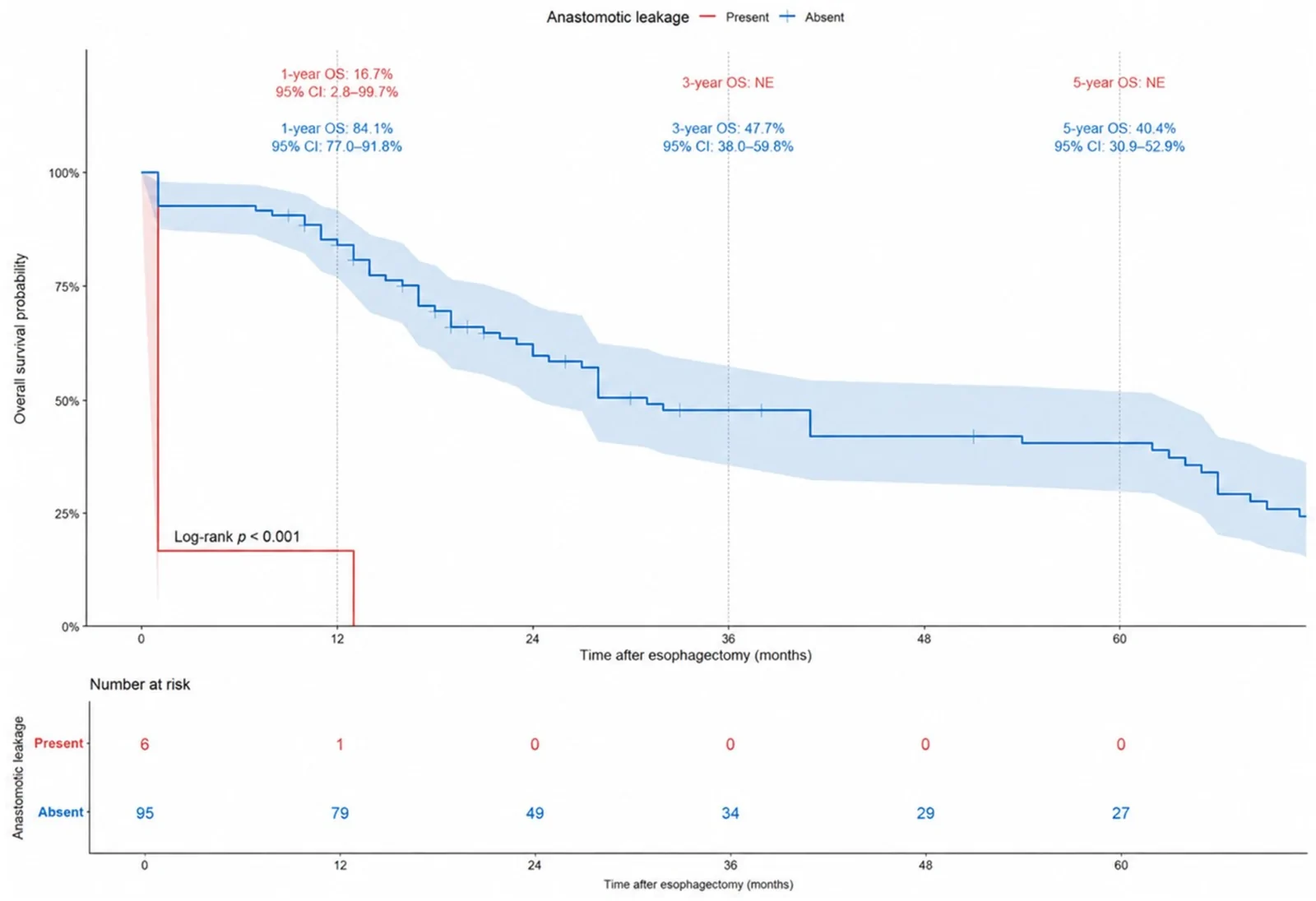
Kaplan–Meier overall survival curves according to anastomotic leakage status. Overall survival differed between patients with and without anastomotic leakage (log-rank *p* < 0.001). Shaded areas represent 95% confidence intervals, and numbers at risk are shown below the survival curves.

**Figure 5:**
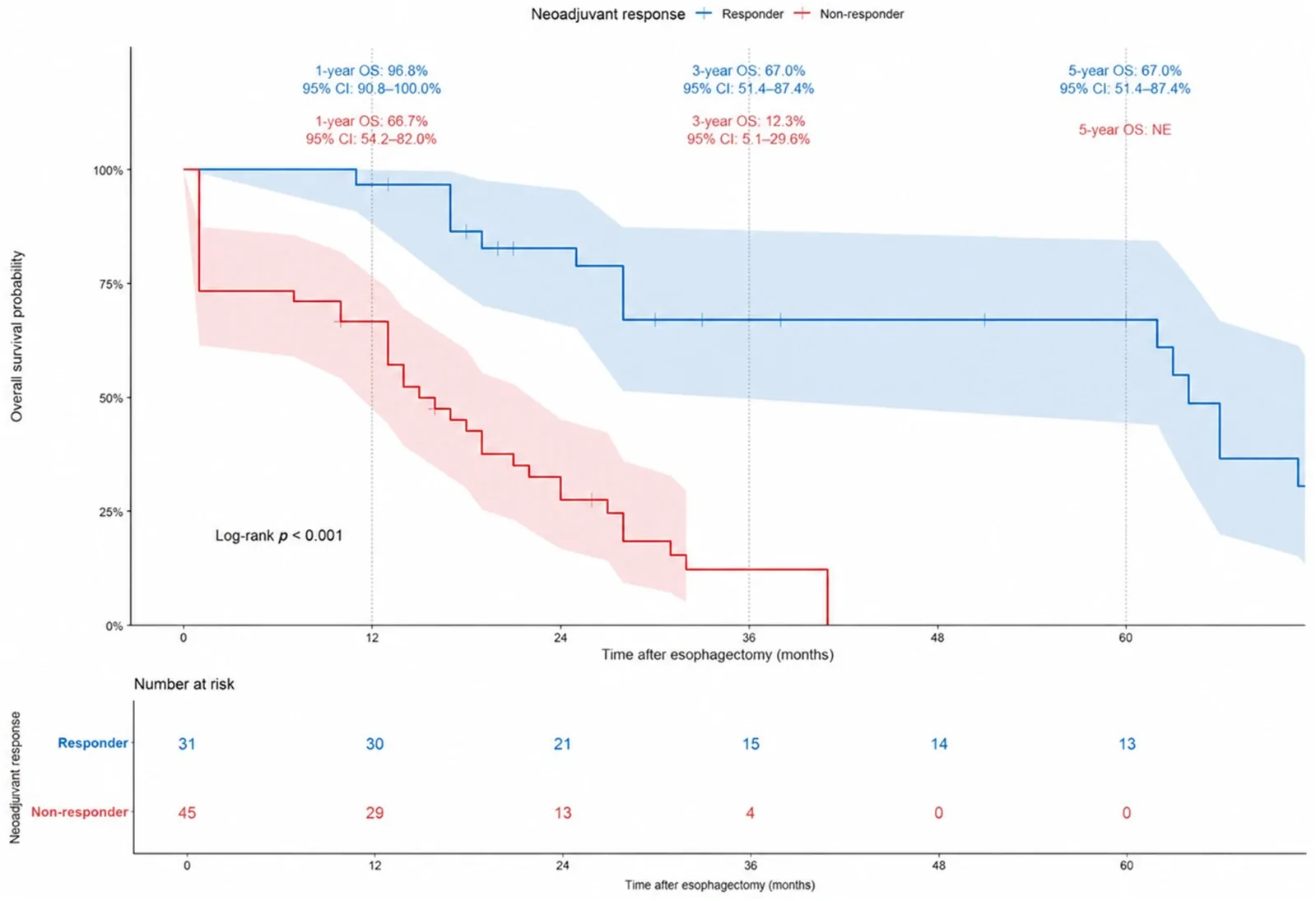
Kaplan–Meier overall survival curves according to pathological response to neoadjuvant therapy. Responders were defined as TRG 1–2 and non-responders as TRG 3–5. Overall survival differed between responders and non-responders (log-rank *p* < 0.001). Shaded areas represent 95% confidence intervals, and numbers at risk are shown below the survival curves.

**Figure 6:**
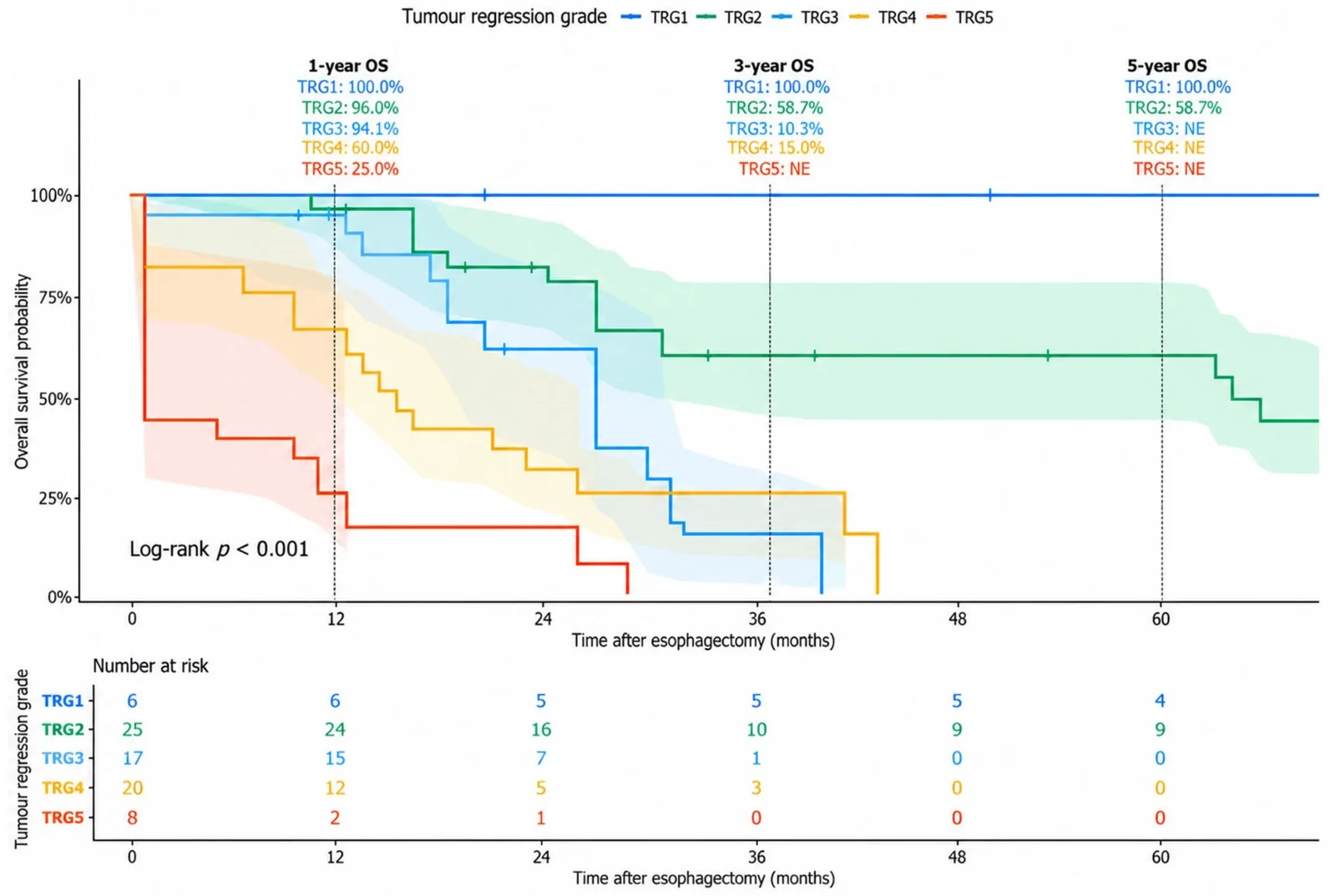
Kaplan–Meier overall survival curves according to tumour regression grade among patients who received neoadjuvant chemotherapy. Overall survival differed significantly across tumour regression grades (log-rank *p* < 0.001), with progressively poorer survival observed with increasing TRG. Shaded areas represent 95% confidence intervals, and numbers at risk are shown below the survival curves.

**Table 1:** Demographic and baseline clinical characteristics according to mortality status.

Variable	Total (n = 101)	90-Day Mortality			Overall Mortality		
		Yes (n = 11)	No (n = 90)	*p*	Yes (n = 74)	No (n = 27)	*p*
Age, n (%)				0.006			0.012
<65	39 (38.6)	0 (0)	39 (43.3)		34 (45.9)	5 (18.5)	
≥65	62 (61.4)	11 (100)	51 (56.7)		40 (54.1)	22 (81.5)	
Gender, n (%)				0.002			0.494
Female	43 (42.6)	0 (0.0)	43 (47.8)		30 (40.5)	13 (48.1)	
Male	58 (57.4)	11 (100.0)	47 (52.2)		44 (59.5)	14 (51.9)	
Smoking Status, n (%)				<0.001			0.004
Yes	50 (49.5)	11 (100.0)	39 (43.3)		43 (58.1)	7 (25.9)	
No	51 (50.5)	0 (0.0)	51 (56.7)		31 (41.9)	20 (74.1)	
ASA Score, n (%)				0.011			0.002
1	1 (1.0)	0 (0.0)	1 (1.1)		0 (0.0)	1 (3.7)	
2	55 (54.5)	3 (27.3)	52 (57.8)		33 (44.6)	22 (81.5)	
3	44 (43.6)	7 (63.6)	37 (41.1)		40 (54.1)	4 (14.8)	
4	1 (1.0)	1 (9.1)	0 (0.0)		1 (1.4)	0 (0.0)	
CEA, n (%)				0.621			0.202
>2.9 ng/mL	53 (52.5)	5 (45.5)	48 (53.3)		36 (48.6)	17 (63.0)	
Normal	48 (47.5)	6 (54.5)	42 (46.7)		38 (51.4)	10 (37.0)	
CA15-3, n (%)				1.000			0.044
>30 U/mL	16 (15.8)	1 (9.1)	15 (16.7)		15 (20.3)	1 (3.7)	
Normal	85 (84.2)	10 (90.9)	75 (83.3)		59 (79.7)	26 (96.3)	
CA19-9, n (%)				0.746			0.694
>37 U/mL	33 (32.7)	4 (36.4)	29 (32.2)		25 (33.8)	8 (29.6)	
Normal	68 (67.3)	7 (63.6)	61 (67.8)		49 (66.2)	19 (70.4)	
Neutrophil count, n (%)				0.362			0.059
>7000/µL	86 (85.1)	11 (100.0)	75 (83.3)		66 (89.2)	20 (74.1)	
Normal	15 (14.9)	0 (0.0)	15 (16.7)		8 (10.8)	7 (25.9)	
Comorbidities n (%)							
DM n (%)				0.592			0.749
Yes	9 (8.9)	0 (0.0)	9 (10.0)		7 (9.5)	2 (7.4)	
No	92 (91.1)	11 (100.0)	81 (90.0)		67 (90.5)	25 (92.6)	
HT n (%)				0.012			0.806
Yes	10 (9.9)	4 (36.4)	6 (6.7)		7 (9.5)	3 (11.1)	
No	91 (90.1)	7 (63.6)	84 (93.3)		67 (90.5)	24 (88.9)	
CAD n (%)				0.016			0.127
Yes	6 (5.9)	3 (27.3)	3 (3.3)		6 (8.1)	0 (0.0)	
No	95 (94.1)	8 (72.7)	87 (96.7)		68 (91.9)	27 (100.0)	

Abbreviations: ASA, American Society of Anesthesiologists; CEA, carcinoembryonic antigen; CA, carbohydrate antigen; DM, diabetes mellitus; HT, hypertension; CAD, coronary artery disease. Categorical variables are presented as n (%). Comparisons were performed using the chi-square test or Fisher’s exact test, as appropriate.

**Table 2:** Surgical, pathological, and oncological characteristics according to mortality status.

Variable	Total n = 101, %	90-Day Mortality			Overall Mortality		
		Yes (n = 11)	No (n = 90)	*p*	Yes (n = 74)	No (n = 27)	*p*
Tumour Localization, n (%)				0.919			0.631
Proximal	1 (1.0)	0 (0.0)	1 (1.1)		1 (1.4)	0 (0.0)	
Mid	11 (10.9)	1 (9.1)	10 (11.1)		7 (9.5)	4 (14.8)	
Distal	89 (88.1)	10 (90.9)	79 (87.8)		66 (89.2)	23 (85.2)	
Histologic Types, n (%)				0.037			0.296
SCC	68 (67.3)	4 (36.4)	64 (71.1)		52 (70.3)	16 (59.3)	
Adenocarcinoma	33 (32.7)	7 (63.6)	26 (28.9)		22 (29.7)	11 (40.7)	
Operation procedures, n (%)				0.181			0.980
Ivor-Lewis	75 (74.3)	10 (90.9)	65 (72.2)		55 (74.3)	20 (74.1)	
McKeown	26 (25.7)	1 (9.1)	25 (27.8)		19 (25.7)	7 (25.9)	
Surgical Approach, n (%)				<0.001			0.027
Minimally invasive	72 (71.3)	0 (0.0)	72 (80.0)		55 (74.3)	17 (63.0)	
Hybrid	12 (11.9)	11 (100.0)	1 (1.1)		11 (14.9)	1 (3.7)	
Open	17 (16.8)	0 (0.0)	17 (18.9)		8 (10.8)	9 (33.3)	
Intraoperative complications, n (%)				<0.001			0.281
Yes	10 (9.9)	7 (63.6)	3 (3.3)		9 (12.2)	1 (3.7)	
No	91 (90.1)	4 (36.4)	87 (96.7)		65 (87.8)	26 (96.3)	
Postoperative complications, n (%)				0.266			0.249
Yes	23 (22.8)	4 (36.4)	19 (21.1)		19 (25.7)	4 (14.8)	
No	78 (77.2)	7 (63.6)	71 (78.9)		55 (74.3)	23 (85.2)	
Anastomotic techniques, n (%)				0.451			0.045
Stapled	80 (79.2)	10 (90.9)	70 (77.8)		55 (74.3)	25 (92.6)	
Hand-sewn	21 (20.8)	1 (9.1)	20 (22.2)		19 (25.7)	2 (7.4)	
Anastomotic Leakage, n (%)				<0.001			0.189
Yes	6 (5.9)	5 (45.5)	1 (1.1)		6 (8.1)	0 (0.0)	
No	95 (94.1)	6 (54.5)	89 (98.9)		68 (91.9)	27 (100.0)	
Anastomotic strictures, n (%)				1.000			0.185
Yes	7 (6.9)	0 (0.0)	7 (7.8)		7 (9.5)	0 (0.0)	
No	94 (93.1)	11 (100.0)	83 (92.2)		67 (90.5)	27 (100.0)	
ypT Stage ^§^, n (%)				<0.001			<0.001
ypT0	6 (7.9)	0 (0.0)	6 (9.2)		1 (1.8)	5 (26.3)	
ypT1a	20 (26.3)	0 (0.0)	20 (30.8)		12 (21.1)	8 (42.1)	
ypT1b	21 (27.6)	0 (0.0)	21 (32.3)		17 (29.8)	4 (21.1)	
ypT2	12 (15.8)	2 (18.2)	10 (15.4)		11 (19.3)	1 (5.3)	
ypT3	10 (13.2)	4 (36.4)	6 (9.2)		10 (17.5)	0 (0.0)	
ypT4a	7 (9.2)	5 (45.5)	2 (3.1)		7 (12.3)	0 (0.0)	
Lymph node Status, n (%)				<0.001			<0.001
Positive	34 (33.7)	11 (100.0)	23 (25.6)		34 (45.9)	0 (0.0)	
Negative	67 (66.3)	0 (0.0)	67 (74.4)		40 (54.1)	27 (100.0)	
Surgical Margin Positivity, n (%)				0.004			0.571
Yes	4 (4.0)	3 (27.3)	1 (1.1)		4 (5.4)	0 (0.0)	
No	97 (96.0)	8 (72.7)	89 (98.9)		70 (94.6)	27 (100.0)	
Neoadjuvant Chemotherapy ^§^, n (%)				0.061			0.493
Yes	76 (75.2)	11 (100.0)	65 (72.2)		57 (77.0)	19 (70.4)	
No	25 (24.8)	0 (0.0)	25 (27.8)		17 (23.0)	8 (29.6)	
Neoadjuvant Chemoradiotherapy, n (%)				0.257			0.329
Yes	53 (52.5)	4 (36.4)	49 (54.4)		41 (55.4)	12 (44.4)	
No	48 (47.5)	7 (63.6)	41 (45.6)		33 (44.6)	15 (55.6)	
Neoadjuvant Response ^§^, n (%)				0.002			<0.001
Responder (TRG 1–2)	31 (40.8)	0 (0.0)	31 (47.7)		17 (29.8)	14 (73.7)	
Non-responder (TRG 3–5)	45 (59.2)	11 (100.0)	34 (52.3)		40 (70.2)	5 (26.3)	
Tumour Regression Grade ^§^, n (%)				<0.001			<0.001
TRG 1	6 (7.9)	0 (0.0)	6 (9.2)		0 (0.0)	6 (31.6)	
TRG 2	25 (32.9)	0 (0.0)	25 (38.5)		17 (29.8)	8 (42.1)	
TRG 3	17 (22.4)	0 (0.0)	17 (26.2)		12 (21.1)	5 (26.3)	
TRG 4	20 (26.3)	5 (45.5)	15 (23.1)		20 (35.1)	0 (0.0)	
TRG 5	8 (10.5)	6 (54.5)	2 (3.1)		8 (14.0)	0 (0.0)	
Locoregional Recurrence, n (%)				0.032			0.080
Yes	28 (27.7)	0 (0.0)	28 (31.1)		24 (32.4)	4 (14.8)	
No	73 (72.3)	11 (100.0)	62 (68.9)		50 (67.6)	23 (85.2)	
Distant Metastasis, n (%)				0.003			0.003
Yes	43 (42.6)	0 (0)	43 (47.8)		38 (51.4)	5 (18.5)	
No	58 (57.4)	11 (100.0)	47 (52.2)		36 (48.6)	22 (81.5)	

Abbreviations: SCC, squamous cell carcino; ^§^ percentages for ypT stage, neoadjuvant response, and tumour regression grade were calculated among patients who received neoadjuvant chemotherapy (n = 76); corresponding mortality subgroup percentages were calculated using the relevant neoadjuvant-treatment subgroup denominators.

**Table 3:** Firth penalized-likelihood logistic regression analysis of 90-day mortality.

Variable	OR (95% CI)	*p*
**Univariable models**		
Age <65 vs. ≥65 years	0.38 (0.07–1.42)	0.157
Male vs. female	21.06 (2.61 –> 1000)	0.001
Smoking: yes vs. no	29.99 (3.71 –> 1000)	<0.001
ASA III–IV vs. I–II	3.46 (0.99–14.82)	0.051
Hypertension: yes vs. no	7.80 (1.82–32.62)	0.007
Coronary artery disease: yes vs. no	10.29 (1.91–56.59)	0.008
CEA elevated vs. normal	0.74 (0.21–2.50)	0.627
CA15-3 elevated vs. normal	0.70 (0.07–3.33)	0.682
CA19-9 elevated vs. normal	1.25 (0.33–4.27)	0.727
Neutrophil count elevated vs. normal	4.72 (0.56–617.75)	0.187
Pathological ypT3–ypT4a vs. ypT0–ypT2 *	25.71 (6.05–153.99)	<0.001
Node-positive vs. node-negative	66.06 (8.06 –> 1000)	<0.001
R1 vs. R0 resection	24.57 (3.57–276.59)	0.001
Intraoperative complication: yes vs. no	41.67 (9.30–233.66)	<0.001
Postoperative complication: yes vs. no	2.20 (0.57–7.70)	0.238
Surgical approach (reference: open)		<0.001 ^†^
Minimally invasive vs. open	0.24 (0.001–45.28)	0.497
Hybrid vs. open	268.33 (19.96 –> 1000)	<0.001
**Bivariable model**		
Node-positive vs. node-negative	103.11 (7.40 –> 1000)	<0.001
Intraoperative complication: yes vs. no	75.58 (7.42 –> 1000)	<0.001

**Abbreviations:** CA, carbohydrate antigen; CEA, carcinoembryonic antigen; CI, confidence interval; OR, odds ratio. Firth penalized likelihood was used because only 11 events occurred and several covariates were completely separated from the outcome, which makes conventional maximum-likelihood estimates infinite or unstable. Odds ratios are accompanied by profile-penalized-likelihood confidence intervals and penalized likelihood-ratio *p* values. An upper limit reported as >1000 indicates that the profile interval was not bounded within a clinically meaningful range. The bivariable model was limited to two covariates in view of the number of events. All estimates should be interpreted as exploratory. * The ypT-stage analysis was restricted to patients who received neoadjuvant therapy (n = 76). † The *p* value for surgical approach represents the global penalized likelihood-ratio test. Open surgery was used as the reference category.

**Table 4:** Multivariable Cox proportional hazards regression analysis of overall survival.

Model	Variable	HR (95% CI)	*p*
**Model 1**	ASA score (III–IV vs. I–II)	1.38 (0.83–2.28)	0.214
	Lymph node status (positive vs. negative)	10.57 (4.78–23.38)	<0.001
**Model 2**	Neoadjuvant response (non-responder vs. responder)	2.96 (1.13–7.73)	0.027
	ypT stage (ypT2–ypT4a vs. ypT0–ypT1b)	1.38 (0.67–2.84)	0.379
	Lymph node status (positive vs. negative)	6.34 (2.60–15.45)	<0.001
	ASA score (III–IV vs. I–II)	1.38 (0.70–2.70)	0.353

**Abbreviations:** HR, hazard ratio; CI, confidence interval; ASA, American Society of Anesthesiologists. Cox proportional hazards regression was used to evaluate overall survival from the date of surgery. Model 1 included the entire cohort (n = 101; 74 deaths) and was stratified by surgical approach because this variable did not satisfy the proportional hazards assumption. Model 2 was restricted to patients who received neoadjuvant chemotherapy (n = 76; 57 deaths). The proportional hazards assumption was satisfied for all covariates included in the final models based on scaled Schoenfeld residuals (global *p* = 0.357 for Model 1 and *p* = 0.341 for Model 2). Harrell’s concordance index was 0.73 for Model 1 and 0.82 for Model 2.

**Table 5:** Kaplan–Meier estimates of overall survival according to selected clinical and pathological variables.

Variable	n	Deaths	Median OS, Months (95% CI)	1-Year OS (%)	3-Year OS (%)	5-Year OS (%)	Log-Rank *p*
Entire cohort	101	74	28 (23–54)	80.1	44.8	38.0	—
Smoking							0.073
Yes	50	43	21 (14–62)	65.8	42.5	38.1	
No	51	31	31 (27–69)	94.0	45.0	35.4	
Lymph node status							<0.001
Positive	34	34	13 (7–17)	52.9	NE	NE	
Negative	67	40	66 (41–73)	93.9	70.4	59.6	
Surgical approach							<0.001
Minimally invasive	72	55	41 (24–63)	90.1	51.4	41.8	
Hybrid	12	11	1 (1–1)	8.3	NE	NE	
Open	17	8	28 (14–NR)	88.2	45.1	45.1	
Anastomotic leakage							<0.001
Present	6	6	1 (1–13)	16.7	NE	NE	
Absent	95	68	31 (24–62)	84.1	47.7	40.4	
ypT stage ^§^							<0.001
ypT0	6	1	NR (11–NR)	85.7	85.7	85.7	
ypT1a	20	12	63 (25–66)	95.0	64.9	64.9	
ypT1b	21	17	28 (18–31)	95.2	18.8	12.5	
ypT2	12	11	17 (1–41)	58.3	31.2	NE	
ypT3	10	10	13 (1–15)	60.0	NE	NE	
ypT4a	7	7	1 (1–13)	28.6	NE	NE	
Neoadjuvant chemotherapy							0.004
Yes	76	57	24 (19–32)	78.9	34.9	27.1	
No	25	17	69 (65–88)	83.5	74.3	69.4	
Neoadjuvant chemoradiotherapy							0.009
Yes	53	41	28 (18–41)	84.9	38.2	26.5	
No	48	33	54 (19–76)	74.8	51.4	48.7	
Neoadjuvant response ^§^							<0.001
Responder	31	17	64 (28–72)	96.8	67.0	67.0	
Non-responder	45	40	16 (13–21)	66.7	12.3	NE	
Tumour regression grade ^§^							<0.001
TRG 1	6	0	NR	100.0	100.0	100.0	
TRG 2	25	17	63 (25–66)	96.0	58.7	58.7	
TRG 3	17	12	28 (18–31)	94.1	10.3	NE	
TRG 4	20	20	14 (1–22)	60.0	15.0	NE	
TRG 5	8	8	1 (1–13)	25.0	NE	NE	

Abbreviations: CI, confidence interval; OS, overall survival; TRG, tumour regression grade; NR, median not reached; NE, not estimable (follow-up shorter than the time point, or no events in the group). Overall survival was measured from the date of surgery to death from any cause; patients alive at last contact were censored. Median survival, 95% confidence intervals and survival rates are Kaplan–Meier estimates, and *p* values are from the log-rank test across all categories of the variable. § Restricted to patients who received neoadjuvant therapy (ypT stage, n = 76; neoadjuvant response and TRG, n = 76). Log-rank tests for trend were significant for both TRG (*p* < 0.001) and ypT stage (*p* < 0.001). ^§^ ypT stage, neoadjuvant response, and tumour regression grade analyses were restricted to patients who received neoadjuvant therapy (n = 76).

## Data Availability

The datasets used and/or analyzed during the current study are available from the corresponding author on reasonable request.

## Supplementary Materials

The following supporting information can be downloaded at https://www.mdpi.com/article/10.3390/jcm15197453/s1, Figure S1: Kaplan-Meier survival curve according to Smoke status; Figure S2: Kaplan-Meier survival curve according to Surgical approach; Figure S3: Kaplan-Meier survival curve according to ypT stage; Figure S4: Kaplan-Meier survival curve according to Neoadjuvant CT; Figure S5: Kaplan-Meier survival curve according to lympnode status among patients who received neoadjuvant chemotherapy.
